# Urinary Cotinine and Nicotine Dependence Levels in Regular Male Electronic Cigarette Users

**DOI:** 10.5152/eurasianjmed.2021.20009

**Published:** 2021-10

**Authors:** Sri Wening Pamungkasningsih, Feni Fitriani Taufik, Erlang Samoedro, Sita Andarini, Agus Dwi Susanto

**Affiliations:** 1 Department of Pulmonolgy and Respiratory Medicine, Universitas Indonesia School of Medicine, Jakarta, Indonesia; Persahabatan Central General Hospital, Jakarta, Indonesia; 2 Dr. H.A. Rotinsulu Lung Hospital, Bandung, Indonesia

**Keywords:** Electronic cigarette, urinary cotinine, nicotine dependence, smoking cessation

## Abstract

**Objective:**

Found in plasma, urine, and saliva, cotinine can be used as a biomarker for nicotine in electronic cigarette (e-cig) users. Nicotine is addictive and causes dependence. Thus, it becomes a problem in smoking cessation programs. This study aimed to determine the relationship between urinary cotinine (UCot) and nicotine dependence levels in regular male e-cig users.

**Materials and Methods:**

This cross-sectional study consecutively included regular male e-cig users and non-smokers. All subjects were interviewed and had their UCot levels measured using an enzyme-linked immunosorbent assay method. The regular e-cig users completed the Penn State Nicotine Dependence Index questionnaire.

**Results:**

This study recruited 71 males aged 18-45 years divided into e-cig users and non-smokers group. The predominating characteristics in e-cig users are as follows: 23 males (67.6%) aged <30 years, the highest education of senior high school 25 (73.5%) and 25 (73.5%) subjects had occupation. The UCot levels among the e-cig users in the non-dependency group were lower than that of the medium-high dependency group (*P =* .008). The median value for UCot level in the regular e-cig users was higher than that of the non-smoker group (276.11 [58.01-284.15] ng/mL vs 5.21 [4.65-23.72] ng/mL, *P <* .001). Factors influencing the UCot levels of the e-cig users were age (*P =* .041), nicotine level of the e-cig liquid (*P =* .013), and the flavor of the e-cig liquid (eg, menthol or non-menthol; *P =* .040).

**Conclusion:**

UCot and nicotine dependence levels in the regular male e-cig users were significantly related. Nicotine dependence was found in 76.5% of the regular male e-cig users. The UCot levels in the e-cig users were significantly higher than in the non-smokers.

## Introduction

The electronic cigarette (e-cig) is a modern innovation of conventional cigarette. Other names are an electronic nicotine delivery system, personal vaporizer, vape pen, e-cigar, e-cigarette, e-cigs, e-hookah, and others. Conventional cigarettes and e-cigs both produce aerosols that distribute nicotine to the lungs, eventually reaching the brain. The aerosol in a conventional cigarette is smoke from the burning process, while an e-cig creates a vapor from the heating process. An e-cig uses a battery to heat a cigarette liquid/e-liquid and turn it into a vapor.^1-^^3^

E-cig vapor does not contain smoke, tar, or carbon monoxide as does conventional cigarette smoke. This creates the perception that e-cigs are relatively safe, but they still contain many toxic substances.^4^ A report from World Health Organization concluded in 2016 that safety of e-cig use has yet to be determined.^5^ The addictive drug in e-cigs is nicotine, which also found in conventional cigarettes. The nicotine level of e-cig liquid ranges from 0 to 36 mg/mL.^6^ The main product of nicotine metabolism is cotinine, which can found in plasma, urine, and saliva. Cotinine, as a biological marker, functioned in examining/evaluating use of nicotine and e-cigs levels.^7^ Most studies on cotinine levels in e-cig smokers have measured from plasma and saliva. Goney et al. conducted the first study on urinary cotinine (UCot) level in e-cig users, in Turkey, in 2016.^3^

A study by Salathe et al. reported that nicotine exposure from e-cigs could cause inflammation, airway hyperactivity, and damage to lung tissue.^8^ Nicotine is also addictive, causing nicotine dependence, and this dependence can be a biological hurdle to quitting smoking.^9^ Different questionnaires can use to assess nicotine dependence in smokers, such as Fagerstrom Test for Nicotine Dependence (FTND), Heaviness of Smoking Index, Wisconsin Inventory of Smoking Dependence Motives and Hooked on Nicotine Checklist. With Penn State Nicotine Dependence Index (PSNDI), Foulds et al. were the first to develop a version of a questionnaire for nicotine dependence to be administered to e-cig users.^10^

A study by Jung et al. reported that UCot levels in conventional smokers were strongly related to nicotine dependence. This result supports the validity of using UCot level in smokers to assess their level of nicotine dependence.^11^ However, a study by Priyonugroho et al. reported that there was no correlation between UCot level and degree of nicotine dependence in cigarette smokers at Balai Besar Rehabilitasi (Rehabilitation Center) Lido, Jawa Barat, Indonesia.^12^ Until the present, there have been no publications on UCot level and nicotine dependence in regular e-cig users in Indonesia. This study aimed to demonstrate differences in UCot levels between regular male e-cig users and male non-smokers, factors affecting UCot levels in regular male e-cig users, nicotine dependence in regular male e-cig users, and relationship between UCot and nicotine dependence levels in regular male e-cig users.

## Materials and Methods

The inclusion criteria were males 18-45 years old, and for the regular e-cig users group, those who had used only e-cigs routinely every day for ≥1 month, with or without a history of conventional cigarette smoking, and for non-smokers group, those with no history of conventional or e-cig use or who had smoked <100 cigarettes over their entire lifetime and had not smoked in the past one month.

The exclusion criteria were the use of nicotine products other than cigarettes (such as chewing tobacco, dipping tobacco, dissolvable tobacco, snuff, forms of nicotine replacement therapy, etc.), use of medications (such as rifampicin, dexamethasone, phenobarbital, artemisinin, oral contraceptives, methoxsalen, tranylcypromine, tryptamine, coumarin, and raloxifene), and having liver or kidney disease. For the e-cig users group, the exclusion criteria included not having used e-cigs in 24 h before the examination and following a smoking cessation program.

This pilot study was cross-sectional and used a consecutive sampling method. The study was conducted from April to October 2018 among a group of regular e-cig users at an e-cig/vape store in Bangka District, Kepulauan Bangka Belitung Province, Indonesia and a group of male non-smokers at Department of Pulmonology and Respiratory Medicine, Universitas Indonesia. The Ethics Committee of the Faculty of Medicine, Universitas Indonesia, gave ethical approval for this study (Document no. 0588/UN2.F1/ETIK/2018). The study subjects were interviewed and then filled out data forms and PSNDI questionnaire, and urine samples were taken and measured for UCot using an enzyme-linked immunosorbent assay (ELISA) method. The UCot levels used in this study were an average value of three samples from each subject and presented in ng/mL. The cotinine level measurement used ELISA method with ELISA cotinine kit catalog number CO096D (Bio-Quant, Inc., San Diego, California). The measurement conducted in the Immunology Laboratory, Department of Pulmonology and Respiratory, Faculty of Medicine, Universitas Indonesia.

Our study used PSNDI questionnaire to assess level of nicotine dependence in regular e-cig users. In our study, we only asked about the dependence level of the subjects toward e-cigs and did not question them regarding their history with conventional cigarette dependence. The PSNDI questionnaire is a modification of several questionnaires used to assess nicotine dependence in conventional or e-cig users. The questionnaire consists of 10 questions with scores for each question. The scores then added to find out level of nicotine dependence. Total number of scores range from 0 to 20 with totals for no nicotine dependence (0-3), low dependence (4-8), medium dependence (9-12), and high dependence (≥13).^8^

### Statistical Analysis

The Statistical Package for the Social Sciences version 20 was used (IBM SPSS Corp.; Armonk, NY, USA). Mann−Whitney nonparametric tests were used to compare UCot level among two groups (e-cig users and non-smokers) also compare UCot level to variables (age, nicotine concentration in e-cig liquid, amount of e-ciq liquid, liquid flavor, and e-cig model). Kruskal–Wallis test were used to compare groups of nicotine dependence to UCOt level. A *P* value < .05 was considered statistically significant.

## Results

### Characteristics of the Subjects

The characteristics of the study subjects in both groups are presented in Table 1. The median age for the regular e-cig user group was 24 (18-45) years old, while the median age for non-smoker group was 32 (22-44) years old. The regular e-cig user group should not have been exposed to conventional cigarette smoke and only exposed to e-cig vapors. In the non-smokers group, there were two subjects (5.4%) who were exposed to conventional cigarette smoke (passive smoker), while 35 subjects were not exposed to either conventional cigarette smoke or e-cig vapors.

The characteristics of e-cig user group, including history of conventional smoking and e-cig use habits, are presented in Table 2. The median time between quitting conventional cigarette smoking and starting e-cig use in this study was 0.5 (0-84) months. As many as 13 former conventional cigarette smokers (50%) directly switched to using e-cigs without a break. The median value for period of e-cig use in this study was 12 (2-60) months. The longest time since last e-cig use was 20 h in one study subject.

### UCot Levels in the Study Subjects

The UCot levels in e-cig users and non-smokers group are presented in Table 3. The UCot level in two subjects in the non-smokers group who exposed to cigarette smoke was 23.6 and 23.72 ng/mL, while the urine cotinine level in one smoker who had last used an e-cig 20 h before the examination was 193.66 ng/mL. The factors affecting the UCot levels analyzed in this study were age, nicotine concentration of the cigarette liquid, the amount of cigarette liquid, the flavor of the cigarette liquid, and the model of e-cig and presented in Table 4. The study results show that there were statistically significant differences in the UCot levels according to age, nicotine concentration, and the flavor of e-cig liquid (*P <* .05) as variables. The median value of the amount of e-cig liquid used by subjects was 5 (2-10) mL/day.

Our study used the PSNDI questionnaire to assess the level of nicotine dependence in regular e-cig users. Table 5 shows the UCot levels in e-cig users and their nicotine dependence level. A Kruskal–Wallis test for data analysis obtained a *P*-value of .015 (*P <* .05). It was statistically significant, implying that there was at least a difference in the UCot levels of the two groups with nicotine dependence. Afterward, we performed a post hoc analysis that found a statistically significant difference between the no dependence group and medium-high dependence group (*P =* .008) and low dependence group and medium-high dependence group (*P =* .029).

## Discussion

### The Characteristics of the Subjects

The regular e-cig users of our study were mostly young. This was probably due to younger people’s interest in e-cigs.^1^ The most common educational level in the e-cig user group was middle education (senior high school graduates). This was probably related to the population socio-demographics of the in Bangka District, where most residents have a middle education. Most of the control group had a high education (college graduates or more) because they were students in the Department of Pulmonology and Respiratory Medicine, Faculty of Medicine, Universitas Indonesia. On the contrary, Hect et al. reported the education for e-cig users as middle education (11.1%) and higher education (88.9%).^13^ This difference was perhaps caused by the socio-demographics of the developed country of the Hect et al. study (United States), while our study held in a developing country (Indonesia). However, similar to this study, Lestari et al. stated that 80% of e-cig users had a middle education and 20% had a high education.^14^ This study held in the same country (Indonesia) as our study, so the socio-demographics were similar.

Most of the subjects in the e-cig user group were employed (73.5%), while the subjects who were not employed were students (26.5%). We connected employment status with current income in relation to the purchasing power for e-cigs. At the time of the study, the lowest price for e-cigs on the market was approximately 300,000 rupiahs, excluding routine e-cig maintenance, such as for the e-cig liquid, atomizer, and more.

### Characteristics of the e-cig User Group

Among the regular e-cig users, there were fewer subjects (23.5%) who had never smoked conventional cigarettes than those with a history of conventional cigarette smoking. This may be related to the fact that conventional cigarettes were available prior to the e-cig, which became available in Indonesia around 2013. Curiosity regarding the e-cig lifestyle caused people who never had smoked conventional cigarettes to become eager to try e-cigs. In a study by Browne et al., only four (0.09%) out of 436 e-cig users did not have a history of smoking conventional cigarettes.^15^ Johnson et al. stated that 19 e-cig users (13.9%) in their study did not have a history of smoking conventional cigarettes.^16^ Our study had a higher percentage of e-cig users who had never smoked conventional cigarettes than either Browne et al. or Johnson et al. This might be related to the young people in our study who were attracted to and then tried e-cigs as a new method for smoking. The median period for e-cig use (12 months) may be related to when the e-cig was first introduced in the Bangka District, approximately three years before the study. Most subjects used e-cigs ≤17.5 h before the urine examination. The average half-life of cotinine is 17.5 h, so this study used 17.5 h as a time limit for prior cigarette smoking.^17^

### UCot Levels in the Subjects

The UCot levels in the e-cig users and non-smokers had a statistically significant difference (*P <* .001). In the e-cig user group, a UCot level of ≥200 ng/mL was seen in 25 subjects (73.5%), while <200 ng/mL was found in nine subjects (26.5%). The study by Lestari et al. of 20 non-routine e-cig users (use three times per week) stated that 88.0% of respondents tested with a positive UCot level (≥200 ng/mL) and 12.0% tested with a negative UCot level (<200 ng/mL). The study used the COT Rapid Test Cassette.^14^ Our study had results similar to the Lestari et al. study in that most of the e-cig users had a UCot level of approximately ≥200 ng/mL. This level may be related to e-cig users having a routine habit.

Hect et al., who studied 28 e-cig users who had used e-cigs at least four times per week during the previous month and had not smoked cigarettes in the previous 2 months, reported that the median value for UCot level was 1880 (1420-2480) ng/mL. The results were higher than in our study possibly because the average nicotine concentration of the e-cig liquid in Hect et al. was higher than that used in our study. A study by Goney et al. of 32 e-cig users found that the average UCot level was 1755 ± 1848 ng/g creatinine. Their measurement of UCot levels used the chromatography method. The UCot level of the e-cig user group did not differ statistically compared to the cigarette smokers.^3^ The results from Goney et al. could not compare with our study because of the difference in the measurement method for the examination of the UCot levels.

A study by Priyonugroho et al. (in the same country with our study) reported the median value for UCot level in cigarette smokers who were participants in a rehabilitation program for addictive substances and given five cigarettes per day as 223.5 (15.8-235) ng/mL.^12^ The median value for UCot in the e-cig users of our study was similar to that of Priyonugroho et al., with a UCot level of 276.11 (58.01-284.15) ng/mL. This contradicts the common opinion that UCot levels are lower in e-cig users than in cigarette smokers. A study by Taufik et al. of 41 cigarette smokers who were treated with shisha cigarettes to measure UCot levels found that the median value prior to shisha use was 159 (7.9-167.2) ng/mL and 30 min after use was 163.2 (13.5-167.3) ng/mL.^18^ The UCot level in our study was higher than with the shisha use by cigarette smokers. This may have been caused by the period after using the shisha and before the test being brief (30 min).

Our study found that the UCot levels in two non-smoker subjects who exposed to cigarette smoke were 23.26 and 23.72 ng/mL. A study by Susanto et al. reported that the median value of the UCot level in children exposed to cigarette smoke was 30.1 ng/mL, while it was 8.45 ng/mL in the non-exposed group.^19^ A different study by Suryatama et al. also reported that the median value for the UCot level in women who exposed to cigarette smoke was 24.65 ng/mL, while it was 7.30 ng/mL in the non-exposed group.^20^ The median value for the UCot level in subjects exposed to cigarette smoke in this study was similar to the median value in previous studies.

### Factors Affecting UCot Level in e-cig Users

In our study, there was a significant statistical difference in the e-cig users group between the UCot levels of those <30 years old and ≥ 30 years old (*P =* .041). However, the study by Priyonugroho et al. stated that there was no correlation between UCot level and age.^12^ The study by Taufik et al. stated that UCot level did not have a statistically significant difference for age in cigarette smokers using shisha.^21^ Nicotine clearance is lower in older age (>65 years old) as compared to young adults. The total clearance of the older group was 23% lower as compared to the younger adults, while renal clearance at an older age was 49% lower as compared to younger adults. The lower volume of nicotine distribution at an older age caused by a decrease in lean body mass.^22^ There were no subjects >65 years old in our study, so there was no tendency for lower cotinine levels correlated to age.

The nicotine concentrations of the e-cig liquids used in our study were only 3 and 6 mg, although there are higher concentrations on the market. The difference in UCot level based on the nicotine concentration in the e-cig liquid was statistically significantly (*P =* .013). These results are in accordance with the theory that cotinine levels in body fluids correlate with the nicotine intake.^23^ Goney et al. also mentioned that there was a positive correlation between UCot level and nicotine concentration in e-cig liquid.^3^

The UCot level for the menthol-flavored liquid had lower levels than non-menthol. As far as we know, at present, there has not been any study of the flavor of e-cig liquid regarding UCot levels. Our study adopted a study by Benowits et al. that claimed that the menthol flavor in cigarettes could inhibit the metabolism of nicotine as compared to cigarettes without menthol flavor.^21^ This may affect the UCot levels of e-cig users.

In our study, the UCot level and e-cig model did not have a statistically significant difference (*P =* .661). This is probably due to the different components of each e-cig, such as battery power, voltage, electrical current, and others. However, the UCot levels in the users of the advanced refillable model tended to be higher than in users of the standard refillable model. Farsalinos et al. stated that plasma nicotine concentrations in e-cig smokers increased by 35-72% with more advanced e-cig models as compared to first-generation models. The latest generation had advantages such as better shape, battery power, ability to control heating time, and others.^24^ The voltage, electrical current, and temperature of the e-cig model affected the composition of the cigarette vapor and later affected the UCot level.^25^

### Relationship between UCot and Nicotine Dependence Level in Regular Male e-cig Users

Our study used the PSNDI to assess the level of nicotine dependence in regular e-cig users. Our study analyzed the data for nicotine dependence by classifying subjects into a no dependence group (score 0-3), low dependence group (score 4-8), and medium-high dependence group (score ≥9). Our study found that there was nicotine dependence in most of the regular e-cig smokers (76.5%), with no dependence on 23.5% of the group. The median score for the e-cig smokers on the PSNDI was 6 (0-16). The existence of nicotine dependence in e-cig users can be a consideration if e-cigs become a method to quit conventional cigarette smoking. The nicotine dependence in e-cig users can be a new form of nicotine dependence in addition to that from conventional cigarettes.^1^

Foulds et al., who used the PSNDI for the first time, stated that the nicotine dependence of conventional cigarette smokers in the past was higher than that of e-cig smokers. Foulds et al. also reported that the average PSNDI score of e-cig users was 8.1 ± 3.5.^10^ Johnson et al. reported that nicotine dependence was higher in e-cig users as compared to conventional smokers.^26^

Our study found a statistically significant result for UCot level and PSNDI questionnaire scores that presented nicotine dependence. This result reflects that there was a relationship between UCot and nicotine dependence levels in e-cig users. As far as we know, to date, there has not been a publication assessing the relationship between UCot and nicotine dependence levels in regular e-cig users. This study adopted a study by Jung et al. stating that UCot levels in cigarette smokers were closely related to nicotine dependence. Jung et al. reported that the UCot level increased along with an increase in the FTND score.^11^

Our study can be considered for the validity of using UCot level in can be used to determining nicotine dependence in regular e-cig users. Our study had a few limitations, such as the small sample size of e-cig users and the one-time data collection in accordance with the cross-sectional study design. In addition, there are many bias factors that could have affected UCot levels in the e-cig users that the study could not minimize, for example, the amount of e-cig liquid, characteristics of vapor topography, and standardization of e-cig components, such as battery power, voltage, and others.

In conclusion, there is a statistically significant relationship between UCot and nicotine dependence levels in regular male e-cig users. As many as 76.5% of male e-cig users had nicotine dependence. The UCot in the regular male e-cig users was significantly higher than that of the non-smokers and significantly influenced by age, nicotine level, and the flavor of the e-cig liquid. As a suggestion, e-cigs cannot use as a method to quit conventional cigarette smoking because they create a new form of nicotine dependence.

**Main Points**

- There was a statistically significant relationship between UCot and nicotine dependence levels in regular male e-cig users.
- There was 76.5% of male e-cig users that had nicotine dependence.
- The UCot in the regular male e-cig users was significantly higher than the non-smokers.
- The UCot in the regular male e-cig users was significantly influenced by age, nicotine level and the flavor of e-cig liquid.

## Figures and Tables

**Table 1.** Characteristics of Subjects

	Total (n = 71)	E-cig users (n = 34)	Non-smokers (n = 37)
Variable	n (%)	n (%)	n (%)
Age			
<30 years old	32 (45.1)	23 (67.6)	9 (24.3)
≥30 years old	39 (54.9)	11 (32.4)	28 (75.7)
Education level			
Middle	28 (39.4)	25 (73.5)	3 (8.1)
High	43 (60.6)	9 (26.5)	34 (91.9)
Employment status			
Employed	60 (84.5)	25 (73.5)	35 (94.6)
Not employed	11 (15.5)	9 (26.5)	2 (5.4)

**Table 2.** Characteristics of the E-cig Users Group

	n (%)
History of conventional cigarette smoking (n = 34)	
Yes	26 (76.5)
No	8 (23.5)
Brinkman index (n = 26)	
Light	15 (44.1)
Moderate	10 (29.4)
Heavy	1 (2.9)
Period between quitting conventional cigarette smoking and starting e-cig using (n = 26)	
≤6 months	23 (67.6)
>6 months	3 (8.8)
Period of e-cig use (n = 34)	
≤6 months	12 (35.3)
>6 months	22 (64.7)
Last e-cig use (n = 34)	
≤17.5 h	33 (97.1)
>17.5 h	1 (2.9)

**Table 3.** UCot Level of Subjects

	N	UCot level (ng/mL)	*P*
		Median (minimum-maximum)	
E-cig user	34	276.11 (58.01-284.15)	<.001^m^
Non-smoker	37	5.21 (4.65-23.72)	

m: Mann−Whitney test

**Table 4.** Factors Affecting UCot Level

	N (%)	UCot level (ng/mL)	*P*
		Median (minimum-maximum)	
Age			
<30years old	23 (67.6)	265.73 (58.01-284.15)	.041^m^
≥30 years old	11 (32.4)	279.35 (148.59-284.15)	
Nicotine concentration of e-cig liquid (mg)			
3	27 (79.4)	264.74 (58.00-284.15)	.013^m^
6	7 (20.6)	282.92 (265.73-284.15)	
Amount of e-cig liquid (mL/day)			
<10 mL/day	21 (61.8)	275.09 (58.01-284.15)	.304^m^
≥10 mL/day	13 (38.2)	279.14 (105.69-284.15)	
Liquid flavor			
Menthol	9 (26.5)	161.02 (106.05-283.39)	.040^m^
Non-menthol	25 (73.5)	277.30 (58.00-284.15)	
E-cig model			
Advanced refillable	32 (94.1)	276.11 (58.01-284.15)	.661^m^
Standard refillable	2 (5.9)	205.98 (132.83-279.14)	

m: Mann−Whitney test.

**Table 5.** UCot and Nicotine Dependence Level

	Total n = 34	UCot Level (ng/mL)	*P*
	n (%)	Median (minimum-maximum)	
No dependence	8 (23.5)	204.62 (58.01-284.15)	.015^kw^
Low dependence	17 (50)	275.09 (72.43-283.78)	
Medium-high dependence	9 (26.5)	280.64 (265.73-284.15)	

kw: Kruskal–Wallis test
